# Rapid degradation of progressive ankylosis protein (ANKH) in craniometaphyseal dysplasia

**DOI:** 10.1038/s41598-018-34157-5

**Published:** 2018-10-24

**Authors:** Jitendra Kanaujiya, Edward Bastow, Raj Luxmi, Zhifang Hao, Dimitrios Zattas, Mark Hochstrasser, Ernst J. Reichenberger, I-Ping Chen

**Affiliations:** 1 Department of Oral Health and Diagnostic Sciences, School of Dental Medicine, University of Connecticut Health, Farmington, CT 06030 United States; 2 Center for Regenerative Medicine and Skeletal Development, Department of Reconstructive Sciences, University of Connecticut Health, Farmington, CT 06030 United States; 3 Program in Structural Biology, Sloan Kettering Institute, New York, NY 10065 United States; 4 Department of Molecular Biophysics and Biochemistry, Department of Molecular, Cellular and Development Biology, Yale University, New Haven, CT 06520 United States

## Abstract

Mutations in the progressive ankylosis protein (NP_473368, human ANKH) cause craniometaphyseal dysplasia (CMD), characterized by progressive thickening of craniofacial bones and widened metaphyses in long bones. The pathogenesis of CMD remains largely unknown, and treatment for CMD is limited to surgical intervention. We have reported that knock-in mice (*Ank*^KI/KI^) carrying a F377del mutation in ANK (NM_020332, mouse ANK) replicate many features of CMD. Interestingly, ablation of the *Ank* gene in *Ank*^KO/KO^ mice also leads to several CMD-like phenotypes. Mutations causing CMD led to decreased steady-state levels of ANK/ANKH protein due to rapid degradation. While wild type (wt) ANK was mostly associated with plasma membranes, endoplasmic reticulum (ER), Golgi apparatus and lysosomes, CMD-linked mutant ANK was aberrantly localized in cytoplasm. Inhibitors of proteasomal degradation significantly restored levels of overexpressed mutant ANK, whereas endogenous CMD-mutant ANK/ANKH levels were more strongly increased by inhibitors of lysosomal degradation. However, these inhibitors do not correct the mislocalization of mutant ANK. Co-expressing wt and CMD-mutant ANK in cells showed that CMD-mutant ANK does not negatively affect wt ANK expression and localization, and vice versa. In conclusion, our finding that CMD mutant ANK/ANKH protein is short-lived and mislocalized in cells may be part of the CMD pathogenesis.

## Introduction

Craniometaphyseal dysplasia (CMD) is a disorder characterized by progressive thickening of craniofacial bones in combination with flaring metaphyses of the long bones^1,2^. Many CMD patients suffer from blindness, deafness, facial palsy and severe headaches due to neuronal compression caused by narrowing of cranial foramina^3–5^. In addition, CMD patients have delayed tooth eruption with mandibulomaxillary hyperostosis^6,7^. There is no effective treatment for CMD except for repetitive surgeries to remove hyperostotic bone for symptom relief. Mutations associated with the autosomal dominant form of CMD have been identified in the progressive ankylosis gene (*ANKH*)^8,9^. Mutations in the *ANKH* gene that are linked to CMD occur mostly within the C-terminal region of the protein resulting in single amino acid substitutions or insertions or in frame deletions^8–11^. Mutations in the N-terminal *ANKH* are most commonly associated with another disorder known as calcium pyrophosphate dehydrate deposition disease (CPPDD)^12^.

The ANKH protein, consisting of 8–12 predicted transmembrane domains with intracellular N- and C-terminus, and its murine homologue ANK transport inorganic pyrophosphate (PPi) into the extracellular matrix and are primarily localized in the plasma membrane^13^. Extracellular PPi serves as an inhibitor of mineralization. ANK also localizes to other cellular compartments, which suggests that ANK may have other functions as well^14,15^. *Ank* knock-out mice (*Ank*^KO/KO^ mice) exhibit some CMD-like phenotypes, such as a narrowed foramen magnum, thickened skull, middle-ear bone fusion and joint stiffness, similar to our previously generated CMD knock-in mice (*Ank*^KI/KI^ mice), where we introduced one of the most common CMD mutations in ANK, a Phe377del mutation^16,17^. *Ank*^KO/KO^ mice do not fully match the phenotype of *Ank*^KI/KI^ mice*. Ank*^KI/KI^ mice replicate additional characteristic skeletal features of CMD patients such as flared femurs and massive jawbones. Osteoblasts and osteoclasts are dysfunctional in *Ank*^KI/KI^ mice^18^. Whereas ANKH mutations lead to the autosomal dominant (AD) form of CMD, most heterozygous *Ank*^+/KI^ mice develop an intermediate CMD-like phenotype with variable expressivity as they age. When compared to 1-year-old *Ank*^+/+^ mice, *Ank*^+/KI^ mice show club-shaped femurs with extensive trabecular bone in diaphyses comparable to *Ank*^KI/KI^ mice^16^. It is not uncommon that mouse models for AD disorders mimic severe phenotypes only in homozygous mice as has been reported for several other disease models^19–21^.

Shared skeletal features between *Ank*^KI/KI^ and *Ank*^KO/KO^ mice and the unique CMD phenotype of *Ank*^KI/KI^ mice indicate that the complex pathogenesis of CMD may be caused by reduced amounts of ANK/ANKH protein as well as by some new function of the mutant protein. In this study, we investigated the expression, localization and protein degradation mechanisms of CMD mutant ANK proteins carrying a deletion of either phenylalanine at position 377 (ΑΝΚ_F377del) or serine at position 375 (ΑΝΚ_S375del). Our results showed rapid degradation of overexpressed CMD-mutant ANK/ANKH protein in transfected cells mainly by the ubiquitin-proteasome pathway, and lysosomal degradation of endogenous ANK/ANKH in mouse and human cells. Although altered functions of CMD-mutant ANK are still unknown, our data show that part of the phenotype seen in *Ank*^KI/KI^ mice is due to decreased amounts of ANK/ANKH protein. These findings are significant not only to better understand the molecular biology of ANK/ANKH protein but also establish a basis for future studies on the pathogenesis and therapeutic strategies of CMD.

## Results

### Decreased expression of ANK/ANKH protein with CMD mutations

**Figure 1:**
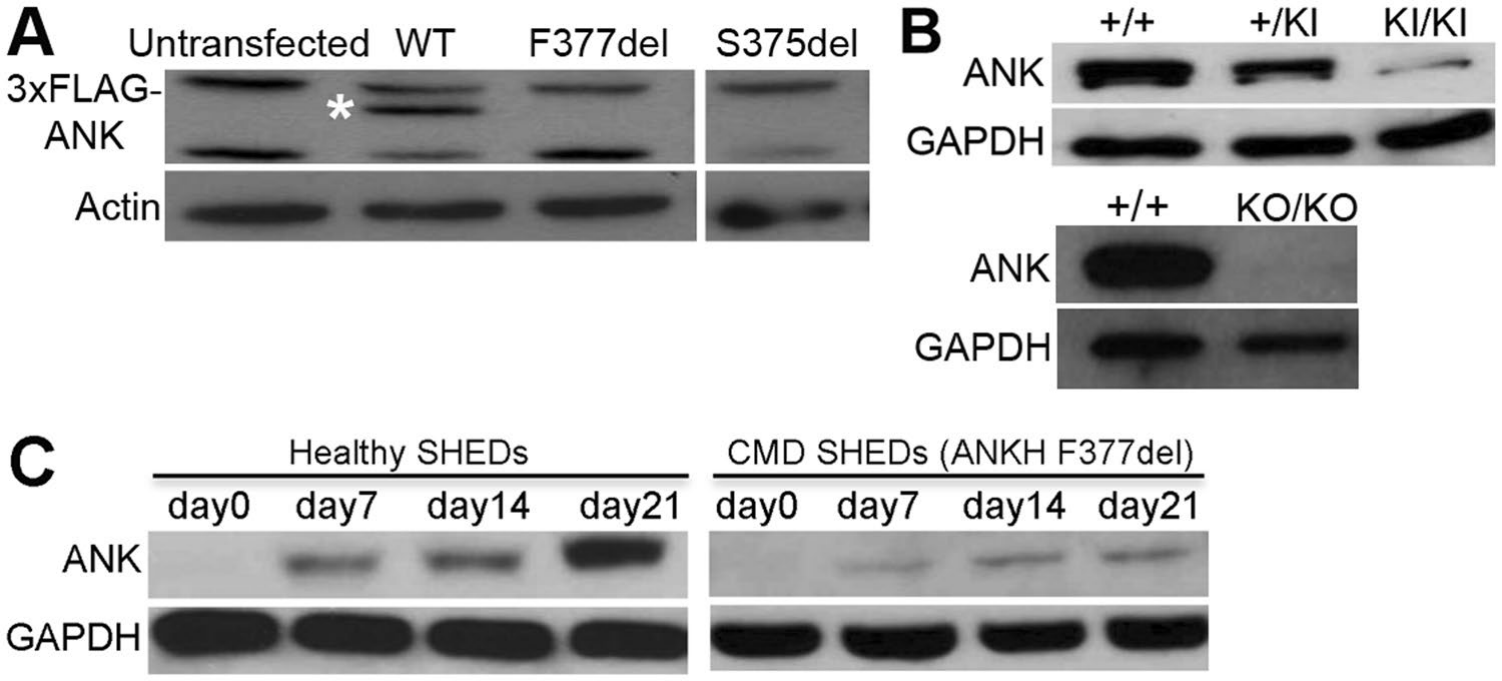
Reduced expression of CMD-mutant ANK/ANKH: Representative images of immunoblotting with FLAG or ANK antibodies of (**A**) ROS cells overexpressing 3xFLAG-tagged wt, -F377del and –S375del ANK; white asterisk indicates 3xFLAG-ANK band immunoblotted with FLAG antibody (**B**) mature osteoclasts from *Ank*^+/+^, *Ank*^+/KI^, *Ank*^KI/KI^ and *Ank*^KO/KO^ BMM cultures (**C**) stem cells from human exfoliated deciduous teeth (SHEDs) differentiated for 0, 7, 14, and 21 days in α-MEM medium supplemented with 50 μg/ml ascorbic acid and 8 mM β-glycerophosphate. Actin and GAPDH are loading controls. Experiments were repeated at least three times.

We examined wild type and CMD-mutant ANK/ANKH protein expression levels in multiple cell types. Protein levels of CMD-mutant ANK were markedly lower than of wild type ANK (ANK_wt) in rat osteosarcoma-derived (ROS) cells over-expressing 3xFLAG-tagged wt, F377del or S375del *Ank* constructs (Fig. 1A). ANK levels were also significantly reduced in bone marrow macrophage (BMM)-derived mature osteoclasts from *Ank*^+/KI^ and *Ank*^KI/KI^ mice compared to *Ank*^+/+^ mice (Fig. 1B). Similarly, ANKH protein levels were strongly reduced in osteoblast-like cells derived from bone explant cultures and in stem cells from human exfoliated deciduous teeth (SHEDs) from CMD patients with ANKH_F377del compared to cells from control individuals (Fig. 1C)^22^. Cell lysate from *Ank*^KO/KO^ osteoclasts served as negative control for antibody binding (Fig. 1B).

### Aberrant intracellular localization of CMD mutant ANK protein

**Figure 2:**
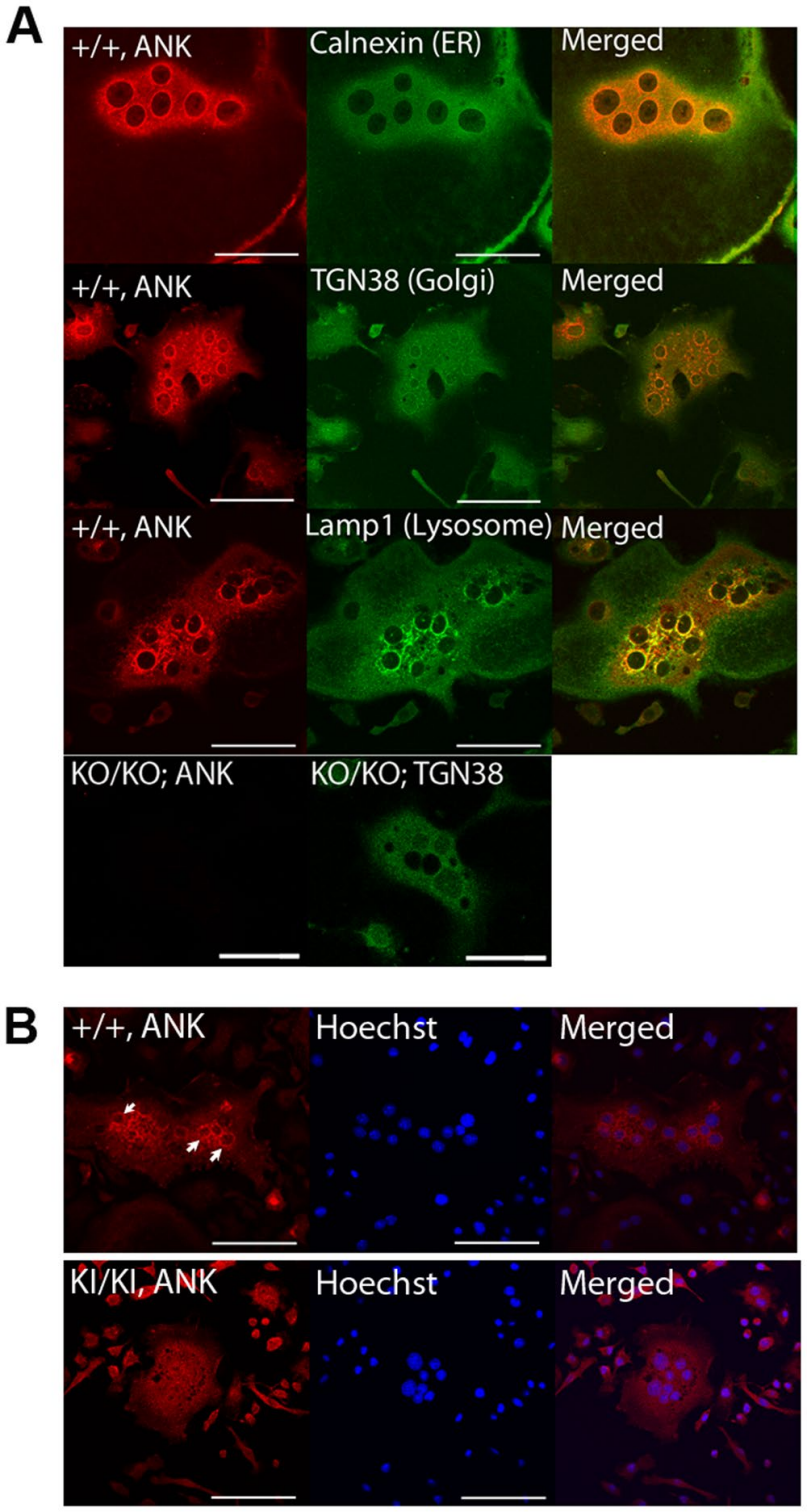
CMD-mutant ANK mislocalizes in multinucleated osteoclasts compared to wt ANK: Representative confocal images of mature osteoclasts with immunostaining showed (**A**) ANK_wt localized to plasma membrane, ER, Golgi and lysosomes in *Ank*^+/+^ OCs, shown by co-staining of ANK (red) with the ER, Golgi and lysosome markers Calnexin, TGN38 and LAMP1 (green), respectively. *Ank*^KO/KO^ OCs served as a negative control; Scale bar = 50 μm (**B**) ANK immunostaining in BMM-derived mature OCs from *Ank*^+/+^ and *Ank*^KI/KI^ mice. Intense expression of ANK_wt associated with membranous organelles (white arrows) was not observed for CMD mutant ANK in *Ank*^KI/KI^ OCs. ANK (red), nuclei staining with Hoechst (blue). Scale bar = 50 μm. Experiments were repeated at least three times.

We next investigated the localization of ANK_wt and ANK_F377del in BMM-derived mature osteoclasts from *Ank*^+/+^ and *Ank*^KI/KI^ mice by immunostaining with antibodies against ANK and several organelle-specific marker proteins (calnexin for ER, TGN38 for Golgi apparatus and LAMP1 for lysosome). Confocal microscopy suggested that wt ANK localized to plasma membrane, ER, Golgi apparatus and lysosomes (Fig. 2A). This distinct expression pattern of wt ANK was not observed in osteoclasts from *Ank*^KI/KI^ BMM cultures where ANK_F377del was mislocalized and dispersed in the cytoplasm (Fig. 2B). The specificity of the ANK antibody was confirmed by the absence of signals in *Ank*^KO/KO^ osteoclasts (Fig. 2A). These data suggest that ANK_F377del may traffic differently compared to ANK_wt and may not be functional.

### Rapid clearance of CMD-mutant ANK/ANKH protein

**Figure 3:**
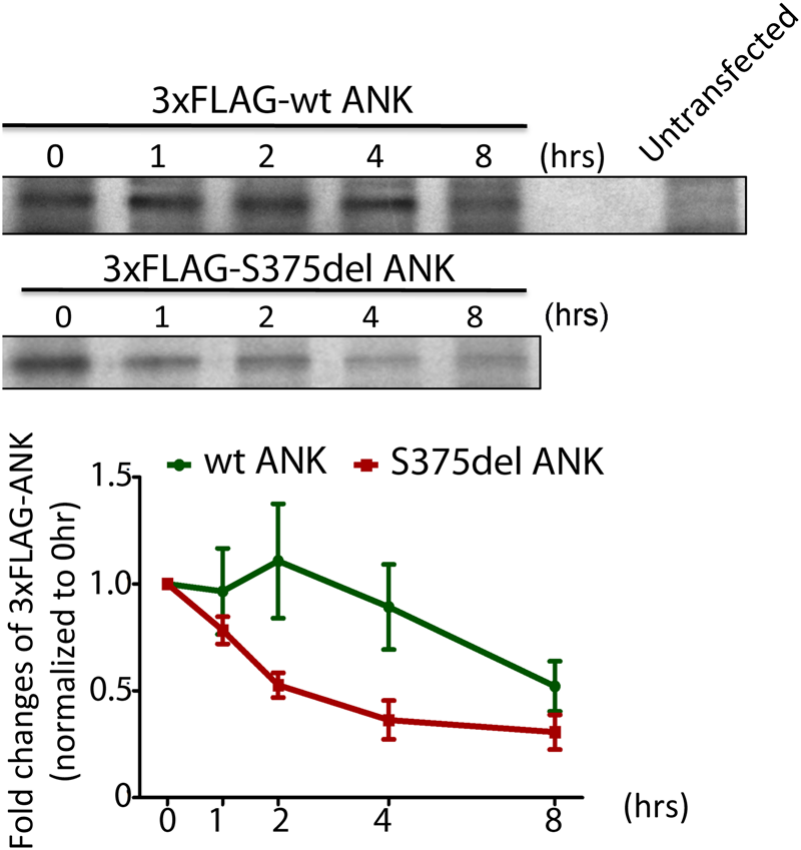
CMD mutant-ANK protein is rapidly degraded compared to wt ANK: Pulse-chase assays of MEFs overexpressing 3xFLAG-wt or -S375del mutant ANK. Cells were labeled with [^35^S]-methionine and chased for 0, 1, 2, 4 and 8 hours. Total cell lysates were subjected to immunoprecipitation with a FLAG antibody and further analyzed by SDS-PAGE and autoradiography. Data averaged from three independent experiments shown in the histogram. Data presented: mean ± S.D.

Despite the decreased protein levels of mutant ANK/ANKH, *Ank* mRNA levels are normal in *Ank*^KI/KI^ mice^18^. Therefore, we examined the stability of wt and CMD mutant ANK protein by pulse-chase assays with [^35^S]-methionine. We transiently expressed 3xFLAG-wt ANK and 3xFLAG-S375del ANK in mouse embryonic fibroblasts (MEFs). Twenty-four hours after transfection the MEFs were incubated in Cys/Met-free media containing ^35^S Met (pulse media). Cells were then chased for 0, 1, 2, 4 and 8 hours with medium containing unlabeled methionine. ANK_S375del was significantly less stable in comparison to ANK_wt (Fig. 3, top panel). Half-lives of ANK_wt and ANK_S375del are approximately 8 and 2 hours, respectively (Fig. 3, bottom panel).

### Increased levels of exogenous CMD mutant ANK by inhibition of ubiquitin-proteasomal degradation

**Figure 4:**
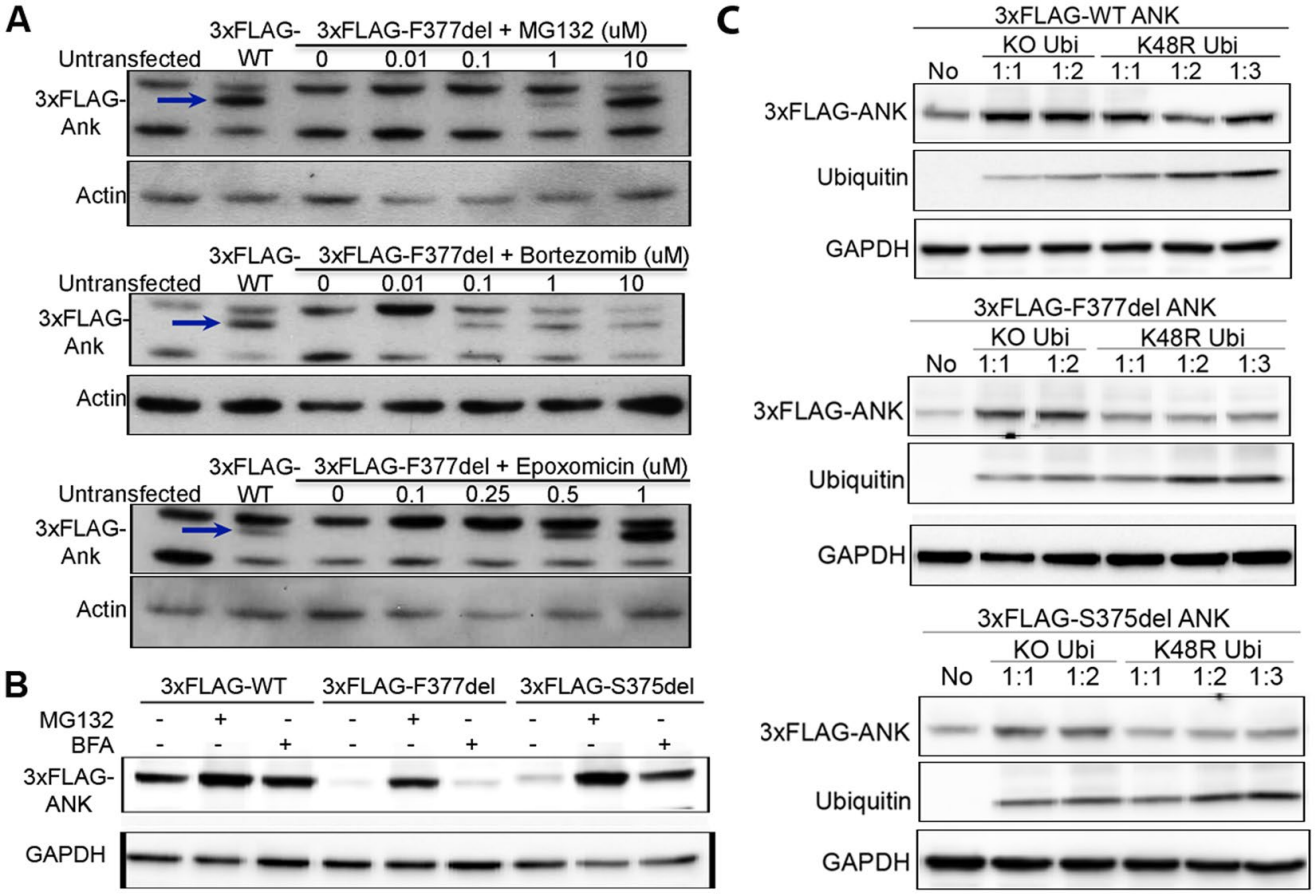
Exogenous CMD mutant-ANK protein is mainly degraded via the ubiquitin-proteasome system (UPS): Immunoblotting with FLAG antibody showed (**A**) inhibitors of proteasomal degradation MG132, bortezomib and epoxomicin rescue 3xFLAG-F377del ANK expression (blue arrows) in transfected ROS cells in a dose-dependent manner (**B**) exogenous CMD mutant ANK (3xFLAG-tagged F377del or -S375del) was restored more by MG132 (5 μM for 9 hours) than by BFA (100 ng/ml for 6 hours) in transfected MEFs. Actin and GAPDH are loading controls. (**C**) Immunoblotting with FLAG and ubiquitin antibodies showed increased steady-state levels of 3xFLAG-wt and mutant ANK in transfected MEFs in the presence of K0 or K48R ubiquitin constructs. GAPDH as loading control. Experiments were repeated at least three times. No: no ubiquitin transfected; Ratio between 3xFLAG-ANK and K0 or K48R ubiquitin was shown as 1:1, 1:2 and 1:3.

Protein clearance occurs mainly via proteasomal or lysosomal degradation. We first examined whether inhibitors of proteasomal degradation can increase CMD-mutant ANK levels. ROS cells overexpressing 3xFLAG-tagged wt, F377del or S375del ANK protein were treated with proteasome inhibitors MG132, bortezomib or epoxomicin for 20 hours. All three inhibitors increased 3xFLAG-tagged F377del or -S375del ANK protein in a dose-dependent manner (Fig. 4A and Supplementary Fig. 1A). Expression of 3xFLAG-tagged wt ANK was also increased by proteasomal inhibitors but not as much as mutant ANK. In contrast, lysosomal inhibitors bafilomycin a1 (BFA; 100 ng/ml for 4 or 9 hours) or chloroquine (CQ; 50 μM for 5 hours) had little to no effect on 3xFLAG-tagged F377del or wt ANK protein levels in ROS cells or MEFs (Fig. 4B and Supplementary Fig. 1B) and only a relatively modest effect on 3xFLAG-tagged S375del ANK amounts in MEFs (Fig. 4B). These findings suggest that overexpressed CMD mutant ANK is predominantly subjected to proteasome-mediated degradation.

High throughput studies showed that ANK_wt protein is polyubiquitinated^23,24^. To investigate whether ubiquitination is required for wt and CMD-mutant ANK/ANKH degradation, we co-transfected MEFs with 3xFLAG-tagged wt or -F377del mutant *Ank* constructs together with HA-tagged lysine-less (K0) or K48R ubiquitin constructs. K0 ubiquitin is a conjugation-deficient ubiquitin mutant that is incapable of forming ubiquitin-ubiquitin isopeptide bonds (all 7 lysines are mutated to arginines). K48-linked chains most commonly target proteins for proteasomal degradation and the K48R mutant specifically disrupts the K48 ubiquitin chain formation. MEFs were transiently transfected with plasmid constructs expressing 3xFLAG-tagged ANK and either K0 or K48R ubiquitin in 1:1, 1:2 or 1:3 ratios. Overexpression of 3xFLAG-tagged ANK and HA-tagged ubiquitin was confirmed by immunoblots with FLAG and HA antibodies, respectively. Disruption of polyubiquitin chain formation with either K0 or K48R ubiquitin constructs resulted in increased accumulation of 3xFLAG-tagged wt, F377del and S375del mutant ANK proteins, but the results with K48R ubiquitin were always more subtle than with K0 ubiquitin (Fig. 4C). These data suggest that both wt and mutant exogenous ANK proteins are degraded via the ubiquitin-proteasome system (UPS), and that non-K48 ubiquitin chains may also contribute to this degradation.

### Heterologously expressed ANK in *Saccharomyces cerevisiae* is degraded via the UPS

**Figure 5:**
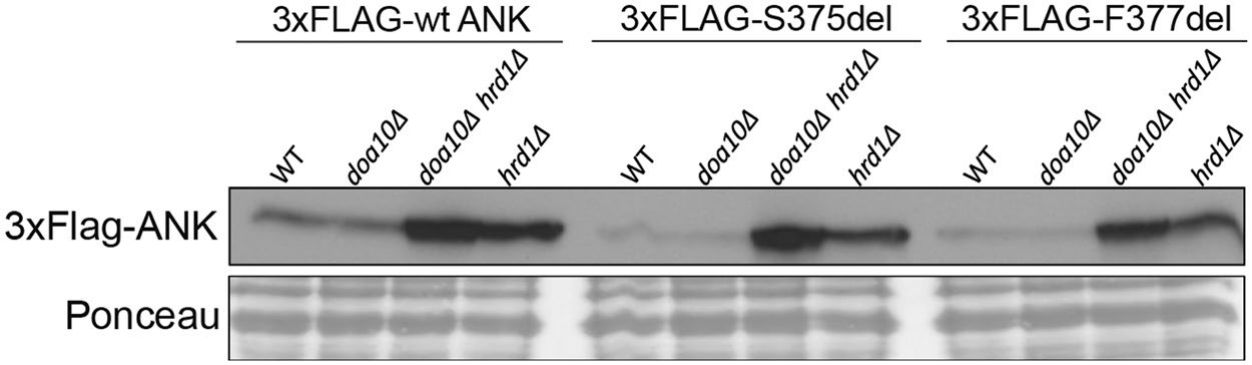
Levels of 3xFLAG-tagged wt, -S375del, and -F377del ANK proteins are increased in yeast strains lacking specific ER-localized E3 ligases. Immunoblot with FLAG antibody showed increased steady-state levels of 3xFLAG-ANK in yeast strains lacking the Hrd1 E3 ligase (*hrd1Δ* and *doa10Δ hrd1Δ* yeast deletion backgrounds), but not in a *doa10Δ* single deletion strain. Ponceau staining served as loading control.

Ubiquitination requires a cascade of E1, E2 and E3 enzymes. The involvement of the UPS in exogenous CMD mutant ANK protein turnover in mammalian cells led us to also test heterologous expression of 3xFLAG-tagged wt, F377del and S375del ANK in wt and E3 ligase-deleted yeast strains. The yeast *S. cerevisiae* has the most fully characterized set of ubiquitination mutants currently available. Because ANK/ANKH proteins are transmembrane proteins that localize at least in part to the ER of mammalian cells (Fig. 2), we hypothesized that the heterologously expressed ANK proteins in yeast might also localize to the ER where they might be targeted for ubiquitin-dependent degradation by the Doa10 and/or Hrd1 E3 ligases. Both the Doa10 and Hrd1 E3 ligases are ER-membrane proteins with important roles in ER-associated degradation (ERAD)^25^. Specifically, we investigated ANK stability in *hrd1Δ, doa10Δ*, or *doa10Δ hrd1Δ* yeast deletion strains, in which protein ubiquitination at the ER is severely compromised. Levels of 3xFLAG-tagged F377del and S375del ANK were strongly increased when expressed in *hrd1Δ* single or *doa10Δ hrd1Δ* double mutant strains in comparison to wt or *doa10Δ* strains (Fig. 5). Steady-state levels of 3xFLAG-tagged wt ANK were higher compared to mutant ANK in wt yeast but were also increased in the *hrd1Δ* or *doa10Δ hrd1Δ* double mutant yeast strains (Fig. 5). These data suggest that mutant ANK proteins are also degraded more rapidly than wt protein when expressed in yeast, as occurs in mammalian cells, and at least a component of this degradation goes through the conserved Hrd1 and, to a lesser extent, Doa10 ERAD pathway.

### Increased levels of endogenous CMD-mutant ANK by inhibition of lysosomal protein degradation

**Figure 6:**
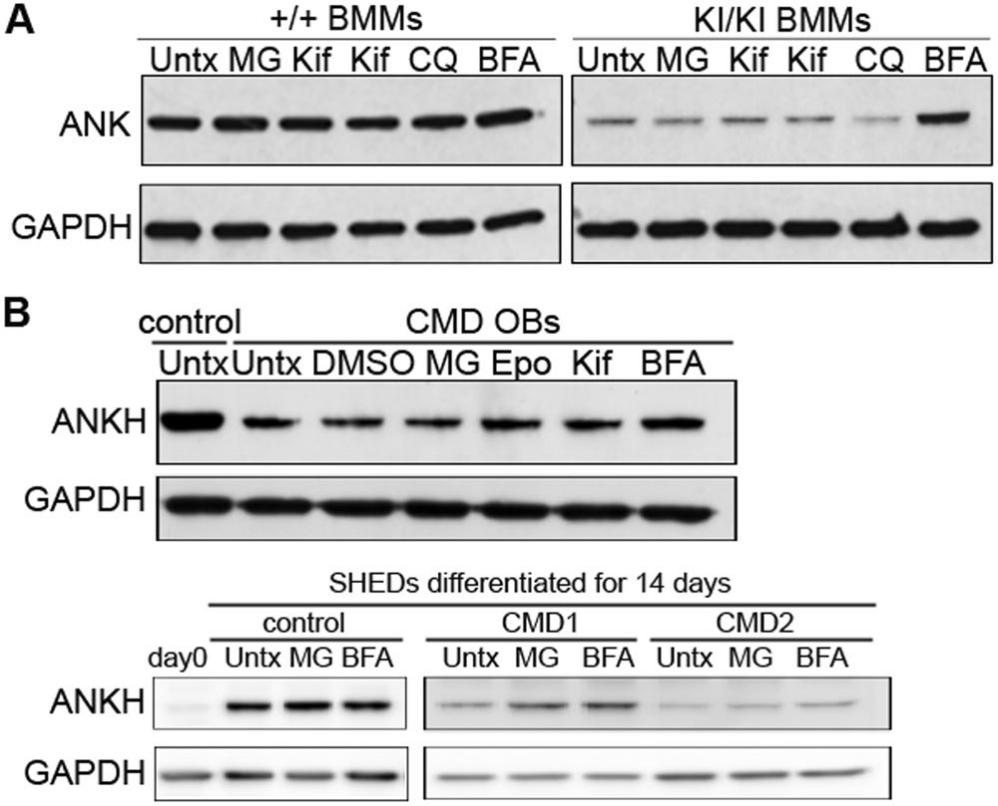
Inhibitors of lysosomal degradation rescue the expression of endogenous CMD mutant ANK: Representative immunoblotting with ANK/ANKH antibody showed that BFA, an inhibitor of lysosomal degradation, rescues ANK_F377del expression better than inhibitors of proteasomal degradation (**A**) in mouse BMM-derived osteoclasts treated with proteasomal inhibitors: MG: 3 μM MG132, Kif: 1 μM (3^rd^ lane) and 3 μM (4^th^ lane) kifunensine, and lysosomal inhibitors: CQ: chloroquine 50 μM, BFA: bafilomycin a1 100 ng/ml (**B**) in osteoblast explant cultures (top panel) of one CMD patient and from SHEDs from one control and two CMD patients (bottom panel) differentiated in ascorbic acid and β-glycerophosphate for 14 days (MG: 5 μM MG132 for 9 hours, Epo: 1 μM epoxomicin for 9 hours, Kif: 1 μM kifunensine for 9 hours, BFA: 100 ng/ml bafilomycin a1 for 6 hours). GAPDH as loading control. Experiments were repeated at least three times, Untx: unreated.

Our observation of increased levels of the overexpressed 3xFLAG-tagged mutant ANK in ROS cells and MEFs in the presence of proteasome inhibitors prompted us to examine whether proteasome inhibitors would rescue the endogenous mutant ANK levels in primary cells. Unexpectedly, we observed only mild increases of mutant protein in primary mouse lung fibroblasts, osteoblasts and osteoclasts (BMMs) after treatment with proteasome inhibitors, despite the accumulation of bulk polyubiquitinated proteins, demonstrating the efficacy of the inhibitor treatment (Supplementary Fig. 2A,B, data not shown). Incubation at low temperatures or with chemical chaperones such as glycerol or high DMSO concentrations have been reported to rescue misfolded proteins^26,27^. We tested these alternative methods of protein rescue but were unable to detect mutant ANK protein in primary cells (Supplementary Fig. 2C and data not shown). We next investigated the effects of lysosomal inhibitors on endogenous wt and mutant ANK expression in BMM-derived osteoclasts from *Ank*^+/+^ and *Ank*^KI/KI^ mice. BFA and chloroquine block autophagic flux by inhibiting the lysosomal Na^+^H^+^ ATPase or by preventing the activity of lysosomal acid proteases via increasing the pH of lysosomes, respectively. We observed that ANK_F377del expression was significantly increased by BFA treatment (fold increase in *Ank*^KI/KI^ BMMs: 3.46 ± 0.54; n = 3) and moderately recovered by chloroquine (fold increase in *Ank*^KI/KI^ BMMs: 1.62 ± 0.69; n = 3) (Fig. 6A and Supplementary Fig. 3). The differences in increasing ANK_F377del levels by BFA and chloroquine were due to the differential efficacy in blocking autophagy shown by the significant accumulation of LC3B-II levels by BFA but less by chloroquine (Supplementary Fig. 3). A moderate increase in ANKH levels was observed after BFA treatment of differentiated cells from bone explant cultures (n = 1) and SHED cultures (n = 2) from healthy controls and patients with the autosomal dominant form of CMD (F377del in ANKH). Increase of ANKH in CMD osteoblast-like cells from bone explant cultures (hOBs), CMD1 SHEDs and CMD2 SHEDs treated with BFA compared to untreated cells were 1.4-, 1.6-, and 1.8-fold, respectively (Fig. 6B). A lesser increase in ANKH levels by BFA treatment is likely due to the fact that human cells are heterozygous for the mutation whereas ANK in mouse cells are homozygous for the ANK_F377del mutation. These results suggest that lysosomal degradation is the preferred route of endogenous mutant ANK degradation, while overexpressed mutant ANK protein is preferentially degraded by proteasomal degradation.

### Mislocalization of mutant ANK is not corrected by proteolysis inhibitors

**Figure 7:**
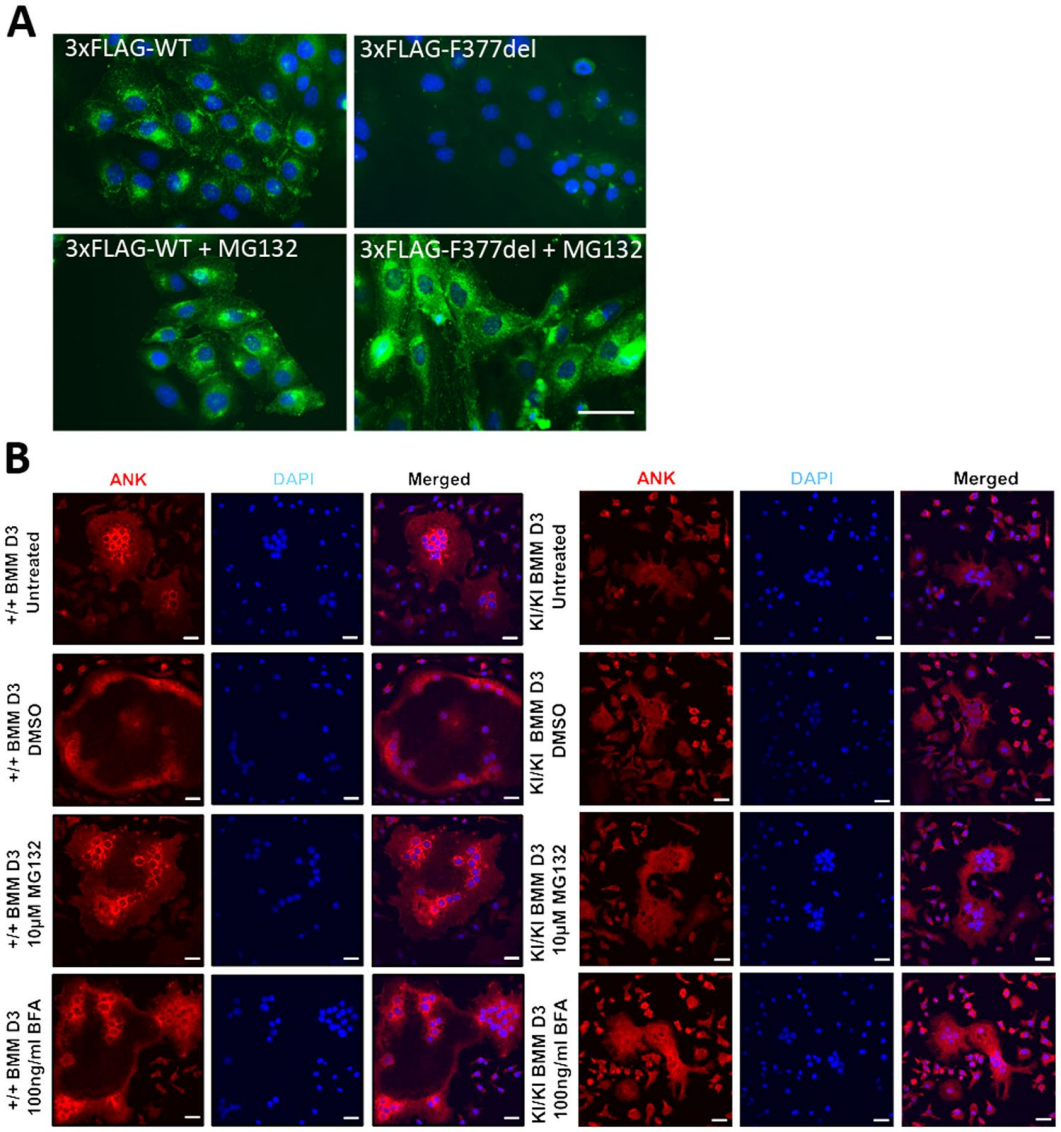
Localization of wt and mutant ANK in the presence of proteasomal and lysosomal inhibitors. (**A**) Immunocytochemistry of ROS cells transfected with 3xFLAG-wt or –F377del ANK in the presence or absence of MG132 using FLAG antibody (green) and Hoechst 33342 stain for nuclei (blue). Scale bar = 50 μm. (**B**) ANK immunocytochemistry with ANK antibodies of BMM-derived osteoclasts untreated or treated with 0.001% DMSO (control), 10 μM MG132 for 9 hrs or 100 ng/ml BFA for 6 hrs. ANK (red), nuclei (blue). Identical exposure time was used for each image. Scale bar = 50 μm.

After finding that proteasome or lysosome inhibitors can reduce degradation of CMD mutant ANK, we explored whether the rescued ANK protein is properly trafficking to specific subcellular compartments. Immunostaining with FLAG antibodies showed 3xFLAG-tagged wt ANK expression around perinuclear regions and on plasma membranes, whereas a weak FLAG signal was observed in cells transfected with 3xFLAG-F377del ANK protein (Fig. 7A, top panel). Strong recovery of the 3xFLAG-F377del ANK protein signal was observed around the nucleus and in the cytoplasm with 1 μM MG132 (Fig. 7A, bottom panel).

We also examined ANK localization in BMMs cultured in medium containing receptor activator of nuclear factor kappa-B ligand (RANKL) to promote osteoclast differentiation for 3 days and treated with MG132 or BFA. Our results showed that wt ANK remains localized to the appropriate subcellular compartments after treating *Ank*^+/+^ BMMs with MG132 or BFA (Fig. 7B, left panel). On the other hand, despite increased intracellular accumulation of ANK signals in *Ank*^KI/KI^ BMMs after inhibitor treatment, especially with BFA, ANK_F377del was not localized to the same cellular compartments as ANK_wt (Fig. 7B, right panel). Stronger ANK intensity in BFA-treated *Ank*^KI/KI^ BMMs is consistent with the above observation that the endogenous ANK_F377del protein is mainly targeted for lysosomal degradation.

### Increasing wt ANK in the presence of CMD mutant ANK

**Figure 8:**
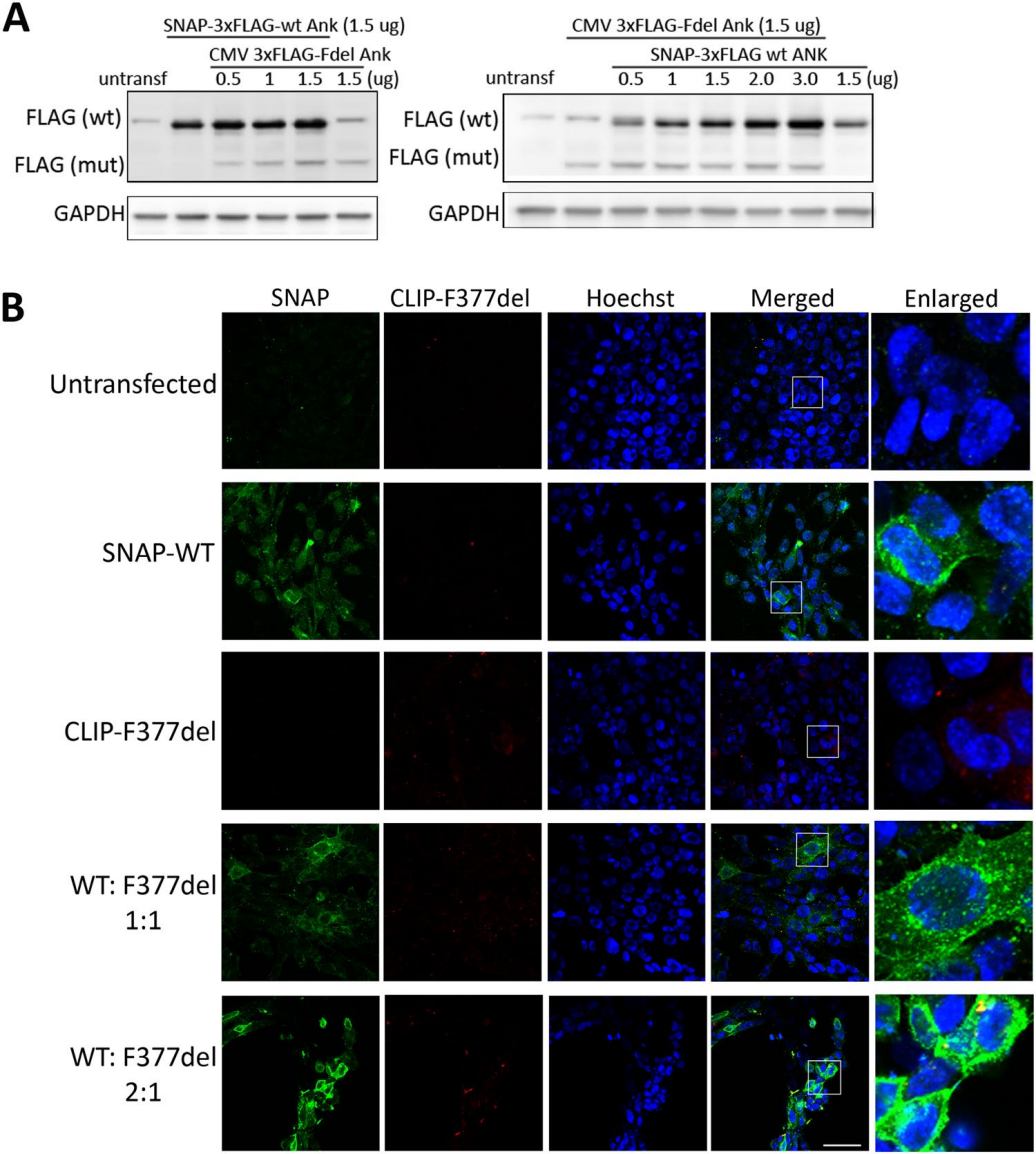
Expression and localization of overexpressed wt ANK in the presence of mutant ANK. (**A**) FLAG immunoblots of MEF cells transfected with SNAP 3xFLAG-tagged wt or/and 3xFLAG–tagged F377del ANK in with various doses. (**B**) Representative confocal images of MEF cells untransfected, transfected with SNAP 3xFLAG-wt alone, CLIP 3xFLAG-F377del ANK alone, SNAP 3xFLAG-wt plus CLIP 3xFLAG-F377del ANK (1:1 ratio) or SNAP 3xFLAG-wt plus CLIP 3xFLAG-F377del ANK (2:1 ratio). Scale bar = 50 μm. All experiments were repeated at least three times. Enlargement of boxed areas from merged images are shown in the right column.

Several ANKH mutations have been identified for the autosomal dominant form of CMD. It is unknown whether mutant ANKH affects wt ANKH protein expression in CMD patients. Since inhibitor treatment did not restore the localization of mutant ANK, we examined whether overexpressing wt ANK can be a strategy to increase the level of normal ANK with appropriate localization in the presence of mutant ANK. If CMD-mutant ANK negatively affects the expression of ANK_wt, we would expect that ANK_wt levels would decrease as mutant ANK levels increase. Therefore we co-transfected MEFs with SNAP-3xFLAG-tagged ANK_wt (size 75 kDa) and 3xFLAG-tagged ANK_F377del (size 54 kDa) at different ratios. MEFs transfected with wt or mutant ANK alone served as controls. We found increasing levels of exogenous ANK_wt protein in correlation with increasing wt *Ank* plasmid transfection despite the presence of mutant *Ank* plasmid. This data suggest that ANK_F377del protein does not negatively affect ANK_wt expression (Fig. 8A).

To study whether ANK_F377del protein affects ANK_wt protein localization, SNAP-tagged wt and CLIP-tagged mutant (F377del) *Ank* plasmids were co-transfected at different ratios in MEFs. Untransfected MEFs and MEFs transfected with wt or mutant *Ank* plasmids alone served as controls. CLIP-tag is a mutant form of the SNAP-tag that uses benzylcytosine as substrate. Our results showed that in the presence of CMD-mutant *Ank*, ANK_wt protein was localized mainly at the plasma membrane and perinuclear area, similar to overexpressing ANK_wt protein alone in the control group (Fig. 8B). Together, these data suggest that CMD mutant ANK does not affect wt ANK expression or localization.

## Discussion

Although the mutations for autosomal dominant CMD in ANKH have been known for more than a decade the molecular mechanisms leading to CMD are still unknown. In this study, we utilized cell lines overexpressing CMD-mutant ANK, cells from a murine CMD model as well as patient cells to better understand the biology of wt and mutant ANK/ANKH proteins. We observed in multiple cell types/tissues from mice, rats, humans and even yeast that CMD-mutant ANK/ANKH was present at a significantly lower level compared to wild type ANK/ANKH. Moreover, the ANK protein bearing CMD mutations was mislocalized and dispearsed in the cytoplasm. This difference in identifiable protein levels appears to be attributable to increased degradation of mutant ANK/ANKH protein compared to wt ANK/ANKH and this observation has been consistent across all cell types investigated. We believe that the reduced levels of functional ANK/ANKH in mutant cells contribute to CMD pathogenesis and may explain shared CMD-like phenotypes between CMD knock-in (*Ank*^KI/KI^) and ANK knock-out (*Ank*^KO/KO^) mice, such as fused middle-ear bones, a narrow foramen magnum and thicker skull bones. The unique CMD phenotypes seen in *Ank*^KI/KI^ but not *Ank*^KO/KO^ mice may be caused by an unknown alteration in function of the ANK_F377del protein as suggested by the difference in subcellular localization.

Proteins exhibit specific structural and chemical features via cotranslational, post-translational modifications and folding to facilitate proper trafficking and for being functional. Genetic mutations may lead to misfolded proteins which can induce several proteolysis mechanisms to prevent further toxic effects. ERAD and unfolded protein response (UPR) are most well-known protein quality control mechanisms of the ER apparatus. Proteins that have mild conformational defects or that are fully assembled after passing through the ER may escape ERAD and may be degraded by post-ER mechanisms, such as Golgi quality control-induced endolysosomal degradation^28–30^. Unfolded cell surface proteins could be recognized by ubiquitin or chaperon proteins and degraded in the lysosomal system via endocytosis^31–34^. Our overexpression system suggests that ANK_wt and ANK_F377del share common degradation mechanisms but ANK_F377del is preferentially degraded. This statement is supported by: (1) overexpressed ANK_wt, ANK_F377del and ANK_S375del were most significantly rescued by inhibitors for proteasomal degradation with increased rescue observed for mutant ANK (Fig. 4B); (2) expression levels of 3xFLAG-tagged wt, ANK_F377del and ANK_S375del protein levels were increased when ubiquitin protein was deficient (Fig. 4C); (3) overexpressed ANK_wt, ANK_F377del and ANK_S375del were all rescued in E3 ligase-deleted mutant yeast strains (Fig. 5). Future studies will need to investigate whether mutant ANK is preferentially recognized by ubiquitin or chaperon proteins leading to rapid degradation.

Degradation mechanisms are utilized differentially for exogenous and endogenous CMD mutant ANK/ANKH proteins. Overexpressed CMD-mutant ANK protein increased strongest when we blocked proteasomal degradation. In contrast, endogenous ANK protein increased more when lysosomal degradation was blocked by BFA treatment. It is, however, possible that partial proteasomal rescue effects for endogenously expressed ANK could not be detected due to limited antibody sensitivity. Similar findings of differential degradation for endogenously and exogenously expressed proteins were reported for the presenilin enhancer 2 homologue (PEN2)^35^. Ubiquitin-dependent proteasomal degradation is likely to be enhanced in transfected cells in order to clear significantly increased amounts of misfolded exogenous mutant protein. It is also possible that a CMV-dependent artifact contributes to the increased levels of wt and mutant ANK after proteasomal inhibitor treatment. It has been reported that proteasomal inhibitors such as lactacystin can non-specifically upregulate the expression of CMV promoter-driven transcription^36–38^. In this current study, we cloned the 3xFLAG-wt, -F377del or -S375del ANK in a CMV promoter-driven vector and overexpressed in ROS cells or MEFs. We observed a higher-fold increase in mutant ANK compared to wt ANK by inhibitors of protein degradation in primary mouse and human cells as well as in transfected cells even when taking into account possible effects of inhibitors on CMV promoter-driven transcripts. The rescue effect of mutant ANK protein is consistent with our finding of faster degradation of CMD mutant ANK in pulse-chase experiments.

ANKH mutations lead to CMD in an autosomal dominant trait. We co-expressed ANK_wt and ANK_F377del to study whether ANK_F377del has dominant negative effects on ANK_wt. In the presence of ANK_F377del, ANK_wt expression level and localization remained unchanged (Fig. 8). Moreover, increasing doses of *Ank_wt* plasmids lead to corresponding increase in ANK_wt protein levels (Fig. 8A, right panel). We suggest that these mutations are likely to cause reduced functionality of ANKH rather than a complete loss-of-function. It is still unclear whether endogenous wt and mutant ANKH in CMD patients are degraded via different mechanisms. In overexpression experiments we differentiated exogenous ANK_wt and ANK_F377del proteins by using different tags, such as SNAP, SNAP-3xFLAG or CLIP. To study degradation mechanisms for endogenous ANKH is challenging because in primary cultures we cannot differentiate between endogenous wt and mutant ANK/ANKH protein. It is also unknown whether increased ANKH levels in CMD patient cells after BFA treatment are primarily due to the recovery of ANKH_wt or ANKH_F377del protein.

Mutations identified in the autosomal dominant form of CMD include a variety of in-frame deletions, substitutions or an insertion in *ANKH*. To date, there are no reports on correlation between mutations and clinical expressivity. In this study, we used two different mutations, S375del and F377del in ANK/ANKH. When overexpressed, both S375del and F377del mutant forms showed reduced protein levels, which were recovered by proteasomal inhibitors. Interestingly, we observed that ANK with the S375del mutation was rescued at a significantly higher level by proteasomal inhibitors than F377del ANK and was more sensitive to BFA treatment (Fig. 4B). It is unknown what causes this difference. Whether different mutations affect the prognosis or progression of CMD differently will need further investigation.

Our data suggest that proteolysis inhibitors should not be considered as a choice to increase functional ANK/ANKH in CMD for several reasons. First, although inhibition of protein degradation can increase mutant ANK/ANKH levels, the recovered amounts of mutant protein were mislocalized to the cytoplasm instead of being membrane-associated, which suggests that its function may be still compromised. Second, protein degradation inhibitors also prevent the degradation of other misfolded proteins, which may have detrimental effects on cells. Furthermore, there are reports that proteasomal inhibitors and the lysosomal inhibitor bafilomycin a1 can suppress osteoclast function^39,40^, although other studies suggest that proteasomal inhibitors may induce osteoclast survival and formation^41^. Previously we reported deficient osteoclastogenesis in the CMD mouse model and in CMD patients, which is in part responsible for hyperostosis of craniofacial bones^18,22^. In addition, bortezomib has been shown to enhance bone formation, partly by modulating runt-related transcription factor 2 (Runx2)^42^. The side effects of proteasomal inhibitors on osteoblasts and osteoclasts may therefore further increase aberrant bone mass in CMD patients.

In summary, ANK/ANKH protein carrying CMD mutations is present only at low levels due to rapid protein degradation. Besides the reduced amounts of ANKH protein, CMD mutations in ANKH are likely to result in yet unknown functional alterations that are responsible for much of the skeletal phenotype. Decreased amounts of functional ANKH and improper subcellular localization are likely to contribute to CMD although these findings do not fully explain the pathogenic mechanisms seen in patients or the mouse model.

## Experimental Procedures

The experiments involving animals were performed in an AAALAC-accredited facility under veterinary supervision. Animal protocol 101425-0819 was approved by the Animal Care Committee of the University of Connecticut Health. All studies involving human protocols were approved by the Institutional Review Board of the University of Connecticut Health (IRB protocol 09–199). All methods were performed in accordance with relevant guidelines and regulations. Informed consent was obtained from all subjects.

### Inhibitors, antibodies and plasmids

Inhibitors for protein degradation and antibodies were purchased from the following suppliers: MG132 (Calbiochem); bortezomib (LC Laboratories); epoxomicin (Calbiochem); bafilomycin a1 (Santa Cruz Biotechnology); ANKH c-terminal antibody (Abgent); beta-actin antibody (Santa Cruz Biotechnology); anti-FLAG (Sigma-Aldrich); Calnexin (Santa Cruz Biotechnology); TGN38 (SeroTech); LAMP1 (Santa Cruz Biotechnology); Ubiquitin, LC3B, HA-Tag (Cell Signaling Technology); horseradish peroxidase-conjugated goat anti-rabbit and goat anti-mouse secondary antibodies (Thermo Scientific); anti-mouse ALEXA 488 secondary antibody (Molecular Probes, Invitrogen). pRK5-HA-Ubiquitin-KO and pRK5-HA-Ubiquitin K48R were purchased from Addgene (plasmid #17608, 17604 and 17603). The ANK polyclonal antibody was generated from rabbits immunized with a KLH conjugated synthetic peptides between 464–492 amino acids from the C-terminal region of ANK/ANKH. The ANK antibody recognizes both wt and mutant ANK/ANKH protein.

### Cloning of p3x-FLAG-wt and mutant Ank in CMV10, pSNAPf, pCLIPf and pRS414 vectors

Wt *Ank* was amplified from mouse cDNA with primers containing Hind III and EcoRI and cloned in p3xFLAG-CMV-10 mammalian expression vector (Sigma-Aldrich). F377del and S375del mutant *Ank* clones were created using a high-fidelity Taq polymerase (New England Biolabs). Wt and mutant *Ank* clones were subcloned N-terminal of pSNAPf and pCLIPf (NEB) mammalian expression vectors using forward primer 5′-GGCGCGCCACCATGG ACTACAAA-3′ containing a restriction site for AscI and a Kozak sequence GCCACC and a reverse primer 5′-TCCGGAATTCCTCATTTT CTTCTCTCATC-3′ containing an EcoRI restriction site without stop codon. These vectors contained SNAP and CLIP-tags. For expression of mouse wt, F377del, and S375del ANK proteins in *S. cerevisiae*, the respective genes containing a 3X FLAG N-terminal sequence were subcloned in a pRS414 vector driven by a MET25 promoter^43^.

### Transfection of rat osteoblast sarcoma (ROS) cells and mouse embryonic fibroblasts (MEFs)

ROS cells were cultured in DMEM/F12 (1:1), L-glutamine, 15 mM HEPES (Gibco Thermo Fisher Scientific), supplemented with 10% fetal bovine serum (Gibco Thermo Fisher Scientific), penicillin and streptomycin. MEFs were cultured in DMEM supplemented with 10% fetal bovine serum, penicillin and streptomycin (Gibco Thermo Fisher Scientific). Cells were passaged when confluent with 0.05% trypsin/EDTA and maintained at 37 °C and 5% CO_2_. ROS cells and MEFs were transfected with wild type *Ank*, mutant *Ank* with deletion of TTC_1130–1132_ (phenylalanine at position 377, F377del) or deletion of TCC_1124–1126_ (serine at position 375, S375del) in p3xFLAG-CMV-10 expression vectors (Sigma-Aldrich) using JetPEI (Polyplus transfection). Stable ROS cell clones expressing the gene construct for wild type and mutant *Ank* were selected by incubating in G418 selection medium. Expression of FLAG-ANK in stably transfected ROS cells was confirmed by PCR followed by immunoblots with FLAG and ANK antibodies. Vectors pRK5-HA-Ubiquitin-KO, pRK5-HA-Ubiquitin K48R, SNAP-3xFLAG-*Ank*, CLIP-3xFLAG-*Ank* were transfected using the same method.

### Mouse calvarial osteoblasts and bone marrow-derived macrophage (BMM) cultures

A knock-in mouse model for craniometaphyseal dysplasia (CMD) expressing a deletion of TTC _1130–1132_ (phenylalanine 377) in *Ank* was generated as described previously^16^. The *Ank* knock-out mouse model was a kind gift from Dr. Kingsley^17^. Mouse calvarial osteoblast cultures were collected from 3- to 5-day-old neonatal mice by a series of enzymatic digestions as described previously^18^. BMM-derived osteoclast cultures were isolated as described previously^18^. Briefly, bone marrow was flushed out from femora and tibia of 7- to 9-week-old male mice and non-adherent cells were collected and purified by Ficoll separation (Lymphoprep, Axis Shield) after 18–24 hours. Cells were seeded at a density of 2 × 10^5^ cells/well on 6-well plates. BMM cultures were treated with M-CSF for 2 days to enrich osteoclast progenitors followed by M-CSF and RANKL (30 ng/ml) for another 3 days to stimulate osteoclast differentiation. Cell lysate was collected for immunoblots.

### Stem cells from human exfoliated deciduous teeth (SHED) and bone explant cultures

Exfoliated deciduous incisors were collected from two normal healthy donors and three CMD patients carrying a F377del mutation in ANKH. SHED cells were isolated as described previously^44^. In brief, pulp was separated from the crown and digested in a mixture of collagenase/dispase solution (Roche) for 1 hour at 37 °C. Cell suspension was filtered through a 70 μm filter (Falcon). Cells were maintained in α-MEM growing medium supplemented with 15% FBS, Pen/Strep and 2 mM glutamine. Once confluent, cells were cultured in growing medium with ascorbic acid (50 μg/ml) and 8 mM β-glycerophosphate (Sigma-Aldrich). Bone explant cultures from one healthy donor and one CMD patient with a F377del mutation in ANKH were performed as previously published^22^.

### Yeast strains

Steady-state levels of mouse 3X-FLAG-wt *Ank*, -F377del, and -S375del mutant ANK proteins in *S. cerevisiae* were investigated in WT yeast (MHY500: *his3-∆200 leu2-3,112 ura3-52 lys2-801 trp1-1*) and various deletion strains lacking the *DOA10* and/or *HRD1* E3 ligase genes (MHY 1685: *his3-∆200 ura3-52 lys2-801 leu2-3,112 trp1-1 doa10∆::HIS3;* MHY 2822: *his3-∆200 ura3-52 lys2-801 leu2-3,112 trp1-1 hrd1∆::LEU2;* and MHY 1703: *his3-∆200 ura3-52 lys2-801 leu2-3,112 trp1-1 doa10∆::HIS3 hrd1∆::LEU2*).

### Heterologous expression and analysis of mouse ANK protein stability in yeast

Yeast cells were transformed with pRS414 MET25 vectors expressing mouse 3X-FLAG-wt, -F377del, or -S375del ANK proteins and selected on solid minimal (SD) medium lacking tryptophan (SD-Trp). Single clonal isolates were selected and grown overnight in liquid SD-Trp media at 30 °C. The next day, each culture was diluted to an OD_600_ of 0.2 and shaken at 30 °C until they reached an OD_600_ range of ~0.6–1 (mid-log). Afterwards, 2.5 OD_600_ units of each culture were collected by centrifugation (2 min, 2,500 × g) and washed once with 1 ml ice-cold dH_2_O. Each cell pellet was resuspended in 1 ml ice-cold dH_2_O followed by the addition of 150 μL of a 2 N NaOH/1 M β-mercaptoethanol solution. Cell suspensions were then incubated on ice for 15 minutes. After this step, 60 μl of 100% trichloroacetic acid solution (~5% final concentration) was added to the suspension and incubated on ice for another hour. Proteins were precipitated by spinning the cell lysates at 21,000 × g for 10 min at 4 °C. The resulting protein pellet was then resuspended in 100 μl of TCA sample buffer (3.5% SDS, 0.5 M DTT, 80 mM Tris, 8 mM EDTA, 15% glycerol, bromophenol blue). Samples were heated at 37 °C for 20 minutes before being loaded on an SDS-PAGE gel. Immunoblot analysis was performed using an anti-FLAG antibody (1:5,000 dilution, F3165, Sigma) followed by incubation with peroxidase-coupled anti-IgG and visualized by Enhanced Chemiluminescence (GE Healthcare).

### Immunoblotting

Cells were lysed from cell culture plates by scraping in the presence of RIPA lysis buffer (150 mM NaCl, 50 mM Tris, 1% NP40, 0.5% deoxycholate and 0.1% SDS) containing 1x HALT protease inhibitor cocktail (Thermo Fisher Scientific). The concentration of protein within the soluble protein lysate was determined by BCA protein assay (Thermo Fisher Scientific). For immunoblotting assays the protein lysates were normalized to total protein concentration and the samples were loaded onto SDS-PAGE gels in 6x loading buffer. Samples were transferred from SDS-PAGE gels onto PVDF membranes (BioRad) using a semi-dry transfer apparatus (BioRad). The membranes were blocked in 5% skim milk Tris-buffered saline/Tween-20 (TBST) overnight and then probed with primary antibody in 1% skim milk in TBST followed by the appropriate horseradish peroxidase conjugated secondary antibody. GAPDH (Santa Cruz Biotechnology) served as internal control. Protein bands were detected by enhanced chemiluminescent detection reagent (Azure Biosystems) and visualized by an Azure c600 imaging system (Azure Biosystems). Densitometric analyses of immunoblots were performed using Image J software. Full-length blots were included in Supplementary information (Supplementary Fig. 4).

### Immunocytochemistry and confocal imaging

ROS cells cultured on glass cover slips were fixed with 2% PFA-PBS, permeabilized with 0.02% Tween20 in PBS for 1 minute and blocked with 5% BSA in PBS for 1 hour at room temperature. Cells were incubated with anti-FLAG antibody (1:4,000) for 2 hours followed by secondary anti-mouse antibody and ALEXA488 (1:1,000) staining for 1 hour. Nuclei were stained with Hoechst 33342 (Molecular Probes, Invitrogen). The coverslips were mounted in Aqua-Poly/Mount (Polysciences Inc.) and images were taken with a Zeiss fluorescence microscope and AxioVision Rel4.8 software.

*Ank*^+/+^ and *Ank*^KI/KI^ BMM cultures were maintained in M-CSF and RANKL (30 ng/ml) for 5 days before using for immunofluorescent staining. Briefly, cells were fixed in 2% paraformaldehyde for 10 minutes at room temperature and permeabilized in 0.2% Triton X-100 for 5 minutes. Cells were blocked in 5% normal goat serum for 1 hour and probed with primary antibody at 1:200 dilution at 4 °C overnight. The next day, cells were incubated with the corresponding secondary antibody at 1:200 dilution for 1 hour at room temperature. Coverslips were mounted in Aqua-Poly/Mount (Polysciences Inc.) and images were taken with a confocal microscope (Zeiss 780) and AxioVision Rel4.8 software.

### [S^35^]-methionine pulse chase assay

Equal numbers of mouse embryonic fibroblasts (MEFs) were transiently transfected with wt or S375del *Ank* in p3xFLAG-CMV-10 expression vectors (Sigma-Aldrich) using Lipofectamine 2000 (Thermo Fisher Scientific) following the manufacturer’s instructions. After 24 hours, overexpressed MEFs were incubated in Cys/Met-free media (pulse media) supplemented with 5% dialyzed FBS for 30 minutes followed by medium containing 500 μCi [^35^S] for 30 minutes, and then chased for 0, 1, 2, 4 and 8 hours with medium containing non-labeled methionine. Cell lysate was immunoprecipitated with FLAG antibody using a Crosslink IP Kit (Thermo Scientific) according to manufacturer’s instructions. In brief, 1 mg of whole cell lysate was pre-cleared against control agarose resin for 1 hour at 4 °C then incubated with anti-FLAG antibodies conjugated to protein A/G Plus agarose overnight at 4 °C with end over end rotation. Samples were washed with IP lysis buffer then eluted in 60 μl elution buffer and 30 μl of the eluted sample was loaded on 10% SDS-PAGE gels for anti-ANK immunoblotting. Gels were fixed, washed and dried. Dried gels were exposed to X-ray film (Blu-Lite^TM^ High Contrast, MTC_TM_ Bio) incubated at −80 °C for one week and developed to visualize the protein bands. The intensity of remaining 3xFLAG-ANK at different time points was quantified by the ImageJ Fiji software and compared to protein levels at time zero.

## Electronic supplementary material

Supplementary Information

## Data Availability

No datasets were generated or analysed during the current study.
