# Supplementary material for: Rapid degradation of progressive ankylosis protein (ANKH) in craniometaphyseal dysplasia
Source: Sci Rep. 2018 Oct 24;8:15710. doi: 10.1038/s41598-018-34157-5 (PMC6200807; doi:10.1038/s41598-018-34157-5)
Supplement: Supplementary file 1 — Supplementary Information [file 41598_2018_34157_MOESM1_ESM.pdf]

Supplementary Information

Rapid degradation of progressive ankylosis protein (ANKH) in craniometaphyseal dysplasia

Jitendra Kanaujiya[1], Edward Bastow[2], Raj Luxmi[1], Zhifang Hao[1], Dimitrios Zattas[3], Mark Hochstrasser[4], Ernst J. Reichenberger[2], I-Ping Chen[1]*

[1]Department of Oral Health and Diagnostic Sciences, School of Dental Medicine, University of Connecticut Health, Farmington, CT 06030, United States
[2]Center for Regenerative Medicine and Skeletal Development, Department of Reconstructive Sciences, University of Connecticut Health, Farmington, CT 06030, United States
[3]Program in Structural Biology, Sloan Kettering Institute, New York, NY 10065, United States
[4]Department of Molecular Biophysics and Biochemistry, Department of Molecular, Cellular and Development Biology, Yale University, New Haven, CT 06520, United States

To whom correspondence should be addressed: Associate Professor I-Ping Chen, Department of Oral Health and Diagnostic Sciences, University of Connecticut Health, 263 Farmington Avenue, Farmington, CT 06030-3705, Telephone: (860) 679-1030; Fax: (860)-679-2910; E-mail: ipchen@uchc.edu

Keywords: progressive ankylosis protein (ANKH), craniometaphyseal dysplasia, ER-associated degradation, autophagy, proteolysis, ubiquitin

Supplementary Figures

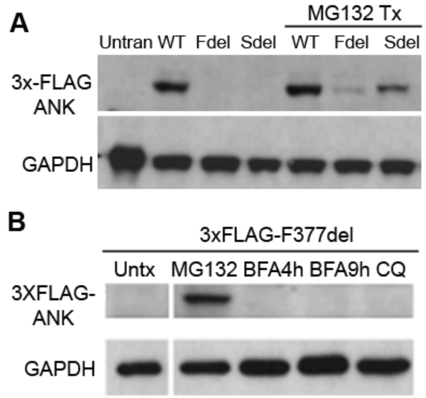

Figure S1. MG132 restores the expression of exogenous CMD mutant ANK. Immunoblotting with FLAG antibody shows that **A)** MG132 rescued both 3xFLAG-tagged F377del and S375del ANK in ROS cells; **B)** 3xFLAG-tagged F377del ANK was mostly rescued by MG132 but not lysosomal inhibitors, BFA (100 ng/ml) for 4 or 9 hours and CQ (50 μM for 5 hours) in transfected ROS cells. GAPDH as loading control.

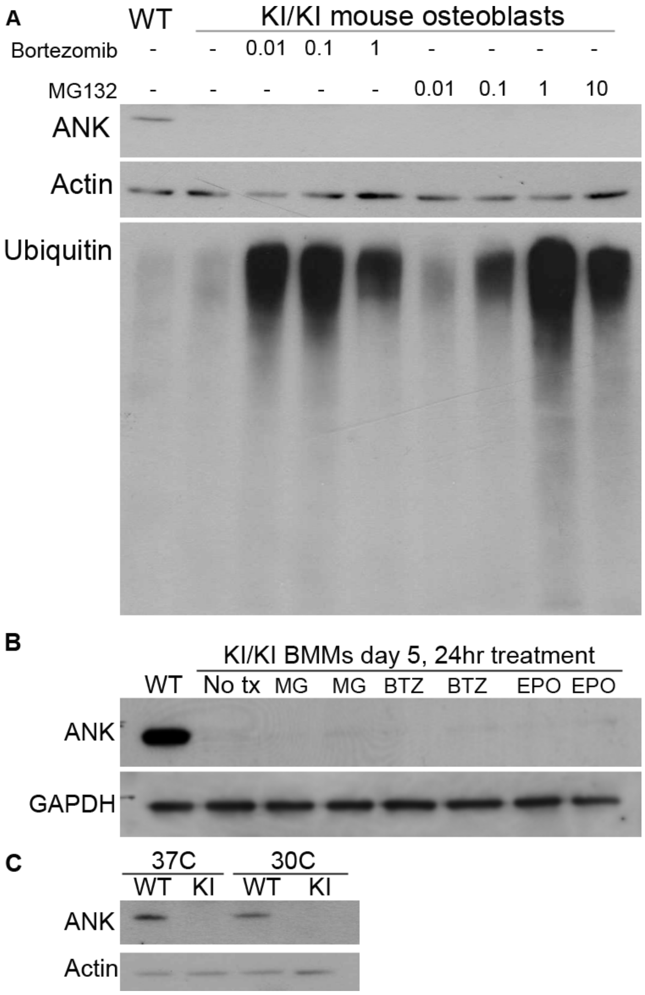

Figure S2. Inhibitors of proteasomal degradation have little effect on recovering the expression of endogenous mutant ANK: no or mild rescue of F377del ANK protein **A)** in *Ank*$^{KI/KI}$ mouse calvarial osteoblasts incubated with increasing concentration of bortezomib or MG132. Total cell lysates were analyzed by immunoblotting for ANK (upper blot). Anti-ubiquitin blotting was used to show blockade of the 26S proteasome, with increasing amount of poly-ubiquitin-tagged proteins observed in bortezomib and MG132 treatment lanes. Actin as loading control. Inhibitor concentration in μM; **B)** in *Ank*$^{KI/KI}$ mouse BMM-derived osteoclasts treated with MG132, bortezomib (BTZ) or epoxomicin (EPO). Concentrations of inhibitors were MG132 10 nM (3rd lane) and 30 nM (4th lane), BTZ 1 nM (5th lane) and 5 nM (6th lane) or EPO 10 nM (7th lane) and 20 nM (8th lane); **C)** in untreated *Ank*$^{KI/KI}$ mouse calvarial osteoblasts cultured at 30°C and 37°C for 24 hours.

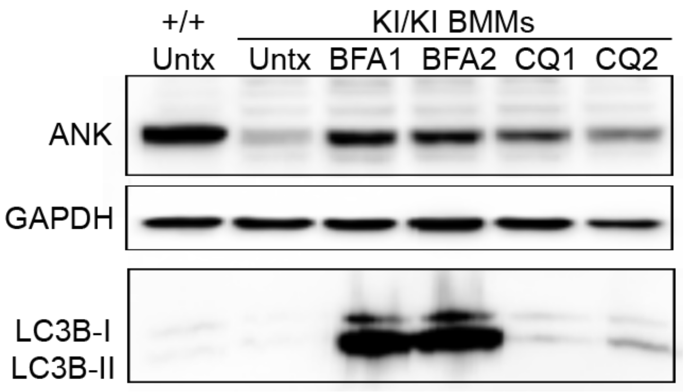

Figure S3. BFA inhibits lysosomal degradation more efficiently than chloroquine in mouse BMMs. Total cell lysates from *Ank*$^{+/+}$ and *Ank*$^{KI/KI}$ BMMs were analyzed by immunoblotting with ANK and LC3B antibodies. Efficiency of blocking autophagic flux was shown by the accumulation of LC3B-II. BFA1: bafilomycin a1 100 ng/ml; BFA2: bafilomycin a1 200 ng/ml; CQ1: chloroquine 25 μM; CQ2: chloroquine 50 μM. GAPDH as loading control.

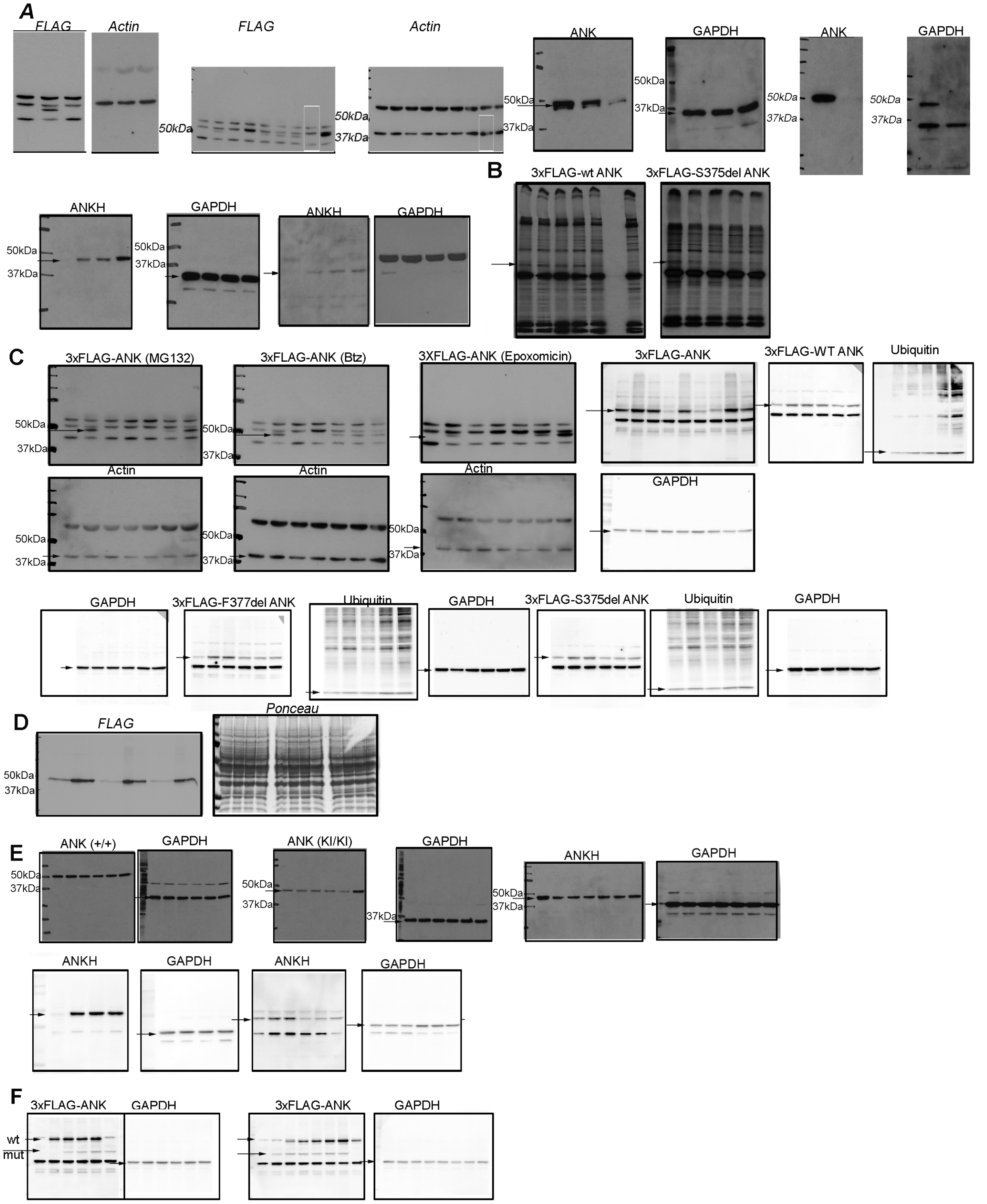

Figure S4. Images of full-length immunoblots. A) Immunoblots shown in Figure 1; **B)** Immunoblots shown in Figure 3; **C)** Immunoblots shown in Figure 4; **D)** Immunoblots shown in Figure 5; **E)**

Immunoblots shown in Figure 6; **F)** Immunoblots shown in Figure 8. Arrows indicate specific signal in each blot.

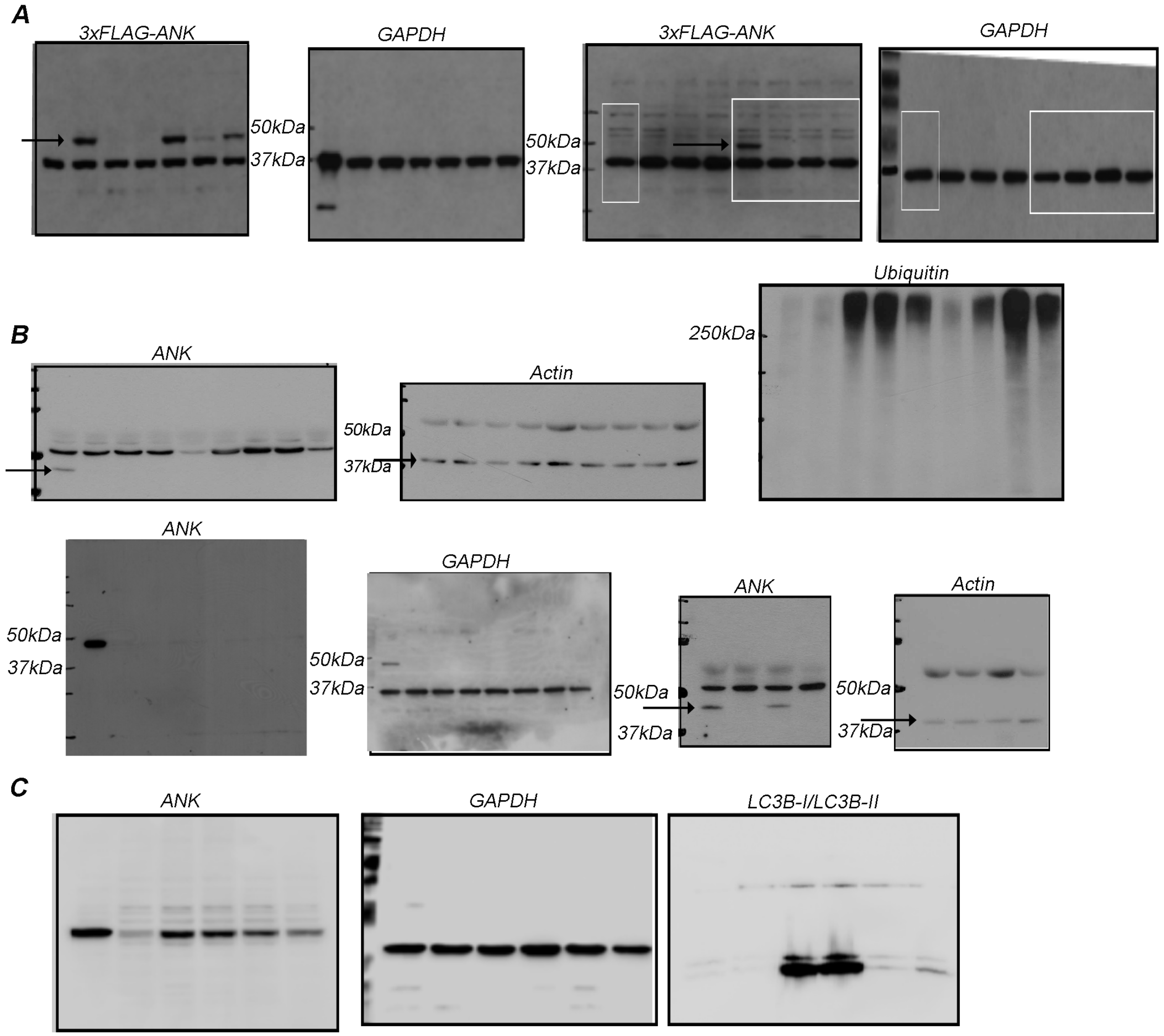

Figure S5. Images of full-length immunoblots. A) Immunoblots shown in Figure S1; **B)** Immunoblots shown in Figure S2; **C)** Immunoblots shown in Figure S3. Arrows indicate specific signal in each blot.
